# Chewing gum and stress reduction

**Published:** 2016-04-24

**Authors:** Andrew P. Smith

**Affiliations:** Centre for Occupational and Health Psychology, School of Psychology, Cardiff University

**Keywords:** stress, chewing gum, mental, health

## Abstract

The behavioral effects of chewing gum have been studied for over 80 years and much of the research has been concerned with increases in alertness and attention. Another line of research has addressed the issue of whether chewing gum reduces stress. This topic has been studied in the laboratory, in epidemiological studies and real-life interventions.

**Relevance for patients:**

The present article briefly reviews the area and makes the case for dissemination of the findings with the aim of reducing stress in the general population and patient groups.

## 1. Introduction

There has been extensive research on chewing gum and behavior [1, 2] and one area that has received considerable attention is chewing gum and stress reduction. Stress is a major issue both for specific clinical groups and the population in general. It is an increasing problem in many specific contexts such as in work and education. Chewing gum is often observed in highly competitive situations (e.g. football managers often chew gum) and anecdotal evidence of stress reduction associated with chewing led to the research described below. A literature search using the terms “Chewing gum and Stress” in the PubMed search engine identified over 80 articles. However, inspection of the papers revealed that only about 20 were relevant, and that most of these had been published in the last 10 years. Some papers also referred to chewing gum and stress from nicotine withdrawal and these are briefly reviewed here.

## 2. Chewing gum, acute stress and anxiety

In a laboratory study, chewing gum was associated with reduced self-reported stress and anxiety following performance of a stressful multi-tasking framework that requires participants to work on multiple tasks at the same time [3]. However, other research that also tested the effects of chewing gum on a multi-tasking framework which also led to increased stress failed to find an effect of chewing gum on reported stress or anxiety [4].

The effect of gum has also been studied for stress induced by the Trier Social Stress Task (TSST). This task involves making a five-minute public presentation and a mental arithmetic exercise. Measures were taken at baseline, before the task, after the task and following a recovery period. Gum reduced self-rated anxiety, and this effect was the greatest for the post-baseline sessions [5]. A later study used the same stress-induction method [6]. Stress was lower for the gum condition following the TSST and post-recovery, although gum did not have an effect on anxiety.

Other research has shown conflicting effects of chewing gum on stress and anxiety. One study [7] failed to find any benefit of chewing gum on self-reported stress following attempts at an insoluble anagram. Another study [8] found that participants reported lower anxiety after chewing non-caffeinated placebo gum (caffeinated gum was also investigated), relative to a no-gum control, although a stressor was not included for this study. This was addressed in later research [9] by testing participants on the same battery of tasks under either quiet or noisy conditions. No effect of chewing gum was observed on a self-reported measure of anxiety, although noise was rated as less annoying during gum conditions.

In summary, experimental research looking at short-term induced stress has shown contradictory findings on self-reported stress and anxiety. The observed effects sizes on self-reported stress and anxiety have been small or moderate. The differences in results may be due to different methods of stress induction being employed in different studies.

## 3. Epidemiological surveys of stress at work and in education

The personal and economic cost of stress is great; statistics published on the HSE website in the UK have indicated that out of 1,152,000 cases of work-related illnesses in the UK in 2010/2011, there were 400,000 cases of stress. Similarly, a survey of undergraduate students by Princeton Review and Wrigley [10] indicated that 85% of students experienced tension and stress at exam times. Abbreviated Progressive Relaxation Training, which involves tensing and relaxing various parts of the body (similar to the process of chewing) has been found to reduce stress [11], and chronic stress has been found to be associated with bruxism [12], so it would seem that clenching the jaw muscles is a natural reaction to stressors that may reduce the intensity of experienced stress. Thirty-six percent of respondents in the Princeton Review and Wrigley survey reported chewing gum while studying, and of these respondents, 41% chewed gum to alleviate stress.

In a survey of workers (N = 2,248), gum chewers reported more exposure to negative characteristics at work (e.g. long or unsociable hours), but fewer participants in this group described themselves as being “extremely stressed” at work [13]. Gum chewers were also less likely to report high levels of life stress than non-chewers. This suggests that chewing gum may ameliorate strong, chronic stress, or perhaps that it is used in an attempt to reduce stress. A second cross-sectional investigation indicated an inverse linear relationship between stress level and amount of habitual gum chewing [14].

## 4. Intervention studies of chewing gum and stress

The cross-sectional investigations described above were followed by a crossover intervention study of chewing gum on stress at work [15]. Participants (university staff; N = 101) were required to chew gum every day for two weeks, and to try to chew gum when they felt stressed. In the non-chewing condition they were required to abstain from gum for two weeks. The chewing gum condition was associated with self-report of lower anxiety and depression, improved mood and lower occupational stress. In line with the survey finding of a dose-response relationship, those who chewed more gum during the intervention experienced a larger positive shift in outcomes. A two-week intervention with a student sample led to reduced stress and enhanced productivity [16].

Other research has carried out interventions with frequent and infrequent gum chewers [17]. Frequent chewers (N = 280) were required to abstain from chewing gum for 3 days and chew gum as normal for another 3 days, and non-regular chewers (N = 212) were required to abstain for 7 days and chew at least three times a day for another 7 days. At the end of each period, participants were questioned about stress, using a simple 5-point scale, and about anxiety levels, using the State-Trait Anxiety Inventory. Abstaining from chewing gum resulted in significant increases in stress and anxiety for both frequent and non-frequent chewers, with reductions in stress and anxiety being observed following periods of chewing.

In summary, chewing gum has been found to reduce self-reported, naturally occurring stress when chewed over a relatively long period of time. Research on the effects of chewing gum on heart rate and levels of cortisol could give a clearer view of whether such effects are visible at a physiological level. Recent studies [2] have examined the effects of chewing gum over a single day and shown reduced benefits compared to the longer interventions. These studies also failed to show effects of chewing on physiological indicators of stress. Many people smoke to reduce stress and to avoid or relieve withdrawal symptoms. Research has shown that smoking status and chewing gum status are correlated, and that chewing gum may reduce withdrawal [18, 19] and not craving for cigarettes.

## 5. Discussion

The research reviewed above suggests that regular chewing of gum may be associated with stress reduction although the acute effects may be more variable [20, 21]. There are plausible biological effects that could underlie such effects. For example, there is evidence that increased glucose metabolism in the rostral medial prefrontal cortex has been associated with lower salivary cortisol [22], which suggests that provision of glucose to relevant brain areas may reduce stress. It is possible that chewing gum may also affect stress through neurotransmission effects. Research [23] has indicated that heightened activity in the ventral prefrontal cortex leads to increased activity of serotonergic neurons in the dorsal raphe nucleus and reduced nociceptive flexion reflex. Overall, it would appear that chewing gum attenuates the sensory processing of external stressors and inhibits the propagation of stress-related information in the brain [24]. The neural mechanisms underlying the stress reducing effects of chewing gum involve the prefrontal cortex which then changes the HPA axis and ANS activity [25, 26].

The majority of research on mental health and chewing gum has been carried out with non-clinical samples. However, if chewing gum can reduce feelings of stress it may attenuate feelings of depression, a stress-related disorder. Strikingly, in a clinical sample of mild-moderately depressed patients, depression was reduced to a greater extent when gum was administered with anti-depressant medication, compared to medication alone [27]. Like many areas there are still a great deal of questions that require fundamental research. However, the benefits of long term chewing on stress reduction suggests that it may be a simple, cost effective method of reducing stress and improving quality of life and well-being. Some may say that it has taken a long time to realize this given that Hollingworth in 1939 described chewing as “a technique of relaxation” [28]. Others may suggest that it is a habit that should be discouraged and that the consequences of poor disposal of the gum may lead to stress.
